# Virtual screening of bioassay data

**DOI:** 10.1186/1758-2946-1-21

**Published:** 2009-12-22

**Authors:** Amanda C Schierz

**Affiliations:** 1 Smart Technology Research Centre, Bournemouth University, Poole House, Talbot Campus, Poole, Dorset, BH12 5BB, UK

## Abstract

**Background:**

There are three main problems associated with the virtual screening of bioassay data. The first is access to freely-available curated data, the second is the number of false positives that occur in the physical primary screening process, and finally the data is highly-imbalanced with a low ratio of Active compounds to Inactive compounds. This paper first discusses these three problems and then a selection of Weka cost-sensitive classifiers (Naive Bayes, SVM, C4.5 and Random Forest) are applied to a variety of bioassay datasets.

**Results:**

Pharmaceutical bioassay data is not readily available to the academic community. The data held at PubChem is not curated and there is a lack of detailed cross-referencing between Primary and Confirmatory screening assays. With regard to the number of false positives that occur in the primary screening process, the analysis carried out has been shallow due to the lack of cross-referencing mentioned above. In six cases found, the average percentage of false positives from the High-Throughput Primary screen is quite high at 64%. For the cost-sensitive classification, Weka's implementations of the Support Vector Machine and C4.5 decision tree learner have performed relatively well. It was also found, that the setting of the Weka cost matrix is dependent on the base classifier used and not solely on the ratio of class imbalance.

**Conclusions:**

Understandably, pharmaceutical data is hard to obtain. However, it would be beneficial to both the pharmaceutical industry and to academics for curated primary screening and corresponding confirmatory data to be provided. Two benefits could be gained by employing virtual screening techniques to bioassay data. First, by reducing the search space of compounds to be screened and secondly, by analysing the false positives that occur in the primary screening process, the technology may be improved. The number of false positives arising from primary screening leads to the issue of whether this type of data should be used for virtual screening. Care when using Weka's cost-sensitive classifiers is needed - across the board misclassification costs based on class ratios should not be used when comparing differing classifiers for the same dataset.

## Background

The drug-development process is both time-consuming and expensive: it takes an average of 15 years and $800 million to bring a drug to the market [1]. The process of discovering a new drug for a particular disease usually involves High-Throughput Screening (HTS), a mixture of robotics, control software, liquid-handlers and optical readers. In HTS, batches of compounds are screened against a biological target (bioassay) to test the compound's ability to bind to the target - if the compound binds then it is an active for that target and known as a *hit*. If this hit is amenable to medicinal chemistry optimization and can be proved to be non-toxic then it may be developed further and become a *lead *for a specific target. Virtual screening is the computational or *in silico *screening of biological compounds and complements the HTS process. It is used to aid the selection of compounds for screening in HTS bioassays or for inclusion in a compound-screening library [2]. Virtual screening can utilise several computational techniques depending on the amount and type of information available about the compounds and the target. Protein-based methods are employed when the 3D structure of the bioassay target is known and computational techniques involve the docking (virtual binding), and subsequent scoring, of candidate ligands (the part of the compound that is capable of binding) to the protein target. Ligand-based approaches are usually used when there are compounds known to be active or inactive for a specific target. If a few active compounds are known then structure-similarity techniques may be used; if the activity of several compounds is known then discriminant analysis techniques, such as machine learning approaches, may be applied. This is achieved by choosing several compounds that have known activity for a specific biological target and building predictive models that can discriminate between the active and inactive compounds. The goal is to then apply these models to several other unscreened compounds so that the compounds most likely to be active may be selected for screening. This is the approach taken in this research.

The major challenge of using machine learning techniques for this type of problem is that the data is highly imbalanced: on average the ratio is 1 active compound to 1000 inactive compounds [3]. This means that standard techniques, which assume equality, are not very effective at building predictive models when there is a low minority class ratio. Any tools employed for Virtual Screening must be able to cope with this imbalance and it is this essential criterion that has led to this investigation of Cost-Sensitive Classifiers (CSC). Virtual screening of imbalanced pharmaceutical data has been carried out before: in one study the classifiers used did not use misclassification costs [4], and in another, the datasets were very small with only a slight imbalance [5]. A recent analysis of PubChem bioassay data using Naive Bayes classifiers has been carried out [6]. However, in their analysis, the number of compounds in the bioassay datasets was reduced so that there was a 1:1 ratio of Active to Inactive compounds. This research is a set of experiments to assess the application of meta-learners included in the Weka suite of machine learning algorithms [7] to a variety of Primary and Confirmatory bioassay datasets. For the rest of this section, we describe the background to this research: the drug-discovery process, bioassay data and cost-sensitive classifiers. We then discuss the methods and results. In the Experimental section, we give descriptions of the datasets, classifiers and data representation. Finally, we discuss and conclude our findings.

### Drug Discovery and Bioassay Data

Drug discovery is the first stage of the drug-development process and is concerned with the selection of compounds to screen and their subsequent screening against a specific biological target. This first stage screening process is known as primary-screening and usually involves the screening of thousands of compounds. One of the problems of the primary-screening process is the number of *false positives *(a compound that has been deemed as active but subsequently turned out to be inactive) that occur. Usually a secondary, or confirmatory, screen of the compound at different doses is required to ascertain its confirmed activity for a specific target. The confirmatory-screening process uses the exact technology as for primary screening but the number of compounds screened is usually significantly smaller: it is usually only the actives from the primary screening process that are used for confirmatory screening.

The main resource for obtaining freely-available bioassay data is the PubChem repository provided by the National Center for Biotechnology Information [8,9]. One of the problems of using the bioassay data from PubChem is that the data is not curated and is potentially erroneous [3,10]. However, there is a lack of publicly-available bioassay data due to the fact that most HTS technology is held at private commercial organisations. In PubChem, there are 506 Primary Screening bioassay results and 858 Confirmatory Screening results (as of November 2009). However, there is no search facility to retrieve the Primary Screening results together with its corresponding Confirmatory Screen (if there is one). Finding corresponding confirmatory bioassays is only achieved by manually going through each primary screen webpage and see if there is one in the *related bioassays *section. The problem is complicated further as sometimes several primary screen bioassay data is used for the one confirmatory screen and vice versa. In database terminology, there is a many-to-many relationship between the 2 types of bioassays. Manually going through each bioassay looking for related bioassays still does not give the complete picture - the bioassay protocol also has to be read. For example, for bioassay AID1919 the Protocol overview states:

*The purpose of this assay is to determine dose response curves for compounds identified as active in a previous set of experiments entitled, "Primary biochemical high throughput screening assay to identify inhibitors of VIM-2 metallo-beta-lactamase" (PubChem AID 1527), and inactive in a set of experiments entitled, "Epi-absorbance primary biochemical high throughput screening assay to identify inhibitors of IMP-1 metallo-beta-lactamase" (PubChem AID 1556)*.

This type of protocol is common in the bioassay data so a lot of data pre-processing has to be carried out to retrieve the relevant compounds from the bioassays. Sometimes finding the relevant confirmed Actives involves manually going through more than one bioassay, for example AID1509 leads to AID1523 which in turn leads to AID1701. Structuring the data this way also hinders the investigation in to why so many compounds end up as being false positives in the primary screening process. Even reading the bioassay protocols does not provide all the necessary information. For example, in primary screening bioassay AID1663 there are 661 bioactive compounds. In confirmatory screen AID1891 the protocol states:

*Counter screen for luciferase inhibitors of Dense Granule Secretion. 20 ul of 1.5 uM ATP (Sigma, #A1852) in PBS is plated in 384-well white assay plates (Aurora, 00030721) and was exposed to the 1584 cherry-picked compounds chosen based on activity of the platelet dense granule release primary screen (AID1663) and structure to compounds with the highest activity, to provide some SAR data*.

Over 900 previously unscreened compounds have been added to the bioactive compounds from the primary screen. This type of bioassay protocol is also common throughout PubChem. Occasionally there are also errors or missing information in the bioassay protocols. For example, in AID688 there are 248 Active compounds but in the confirmatory screen AID792 it states

*The HTS has been reported earlier (AID 688). Here we report the follow-up dose-response testing on the 448 compounds identified as hits in the HTS*.

Other bioassays also contain incorrect information. In AID530, the Data Activity Table is contradictory. According to the main bioassay description, 10,014 compounds were screened with 34 Actives, 9066 Inactives and 1136 Inconclusive compounds. This adds up to 10,236 compounds. When looking at the Data Activity table, the figures are 34 Actives, 9066 Inactives and 222 Discrepant compounds. This adds up to 9322 compounds even though it states that 10,014 compounds were tested. If you download the AID530 Activity information in CSV format, the figures are different from both of these - there are 22 labelled as Active, 8866 as Inactive, 931 as Inconclusive and 195 as Discrepant, which does total the original figure of 10,014.

Out of 250 manually searched confirmatory screening bioassays, only six had good links to the primary screen. However, four of these still had some compounds either added or removed without a detailed explanation why. Table 1 shows a summary of the False Positives that have occurred in the HTS Primary Screen. The table shows the number of Actives founds in the primary screen (PS), the number of compounds tested in the confirmatory screen (CS), the number of Actives in the confirmatory screen and the percentage of false positives from the primary screen.

**Table 1:** Summary of Primary Screen false positives

Primary (PS)	Confirmatory (CS)	PS Actives	CS Tested	CS Actives	False Positive %
AID604	AID644	212	206	67	65.57%
AID1284	AID746	366	362	57	83.33%
AID439	AID373	62	69	13	90.32%
AID721	AID687	94	94	21	77.66%
AID561	AID611	278	273	195	28.06%
AID525	AID600	359	359	213	40.67%

Though a detailed analysis could not be carried out due to the lack of information provided, these false positive rates are quite high (average 64%) and possibly suggest that primary screening data should not be used for virtual screening. For AID688, mentioned above for the cross-referencing error, there was a 100% false positive rate according to the confirmatory screen AID792. These figures have not been included in Table 1 in case they are also erroneous.

## Methods

### Bioassay Datasets

A variety of datasets have been chosen for this study. Unfortunately due to computer memory limitations (Weka can only utilise 2 gigabytes of heap space for Windows systems), only small to medium datasets have been selected. However, the datasets are from the differing types of screening that can be performed using HTS technology (both primary and confirmatory screening) and they have varying sizes and minority classes. 21 datasets were created from the screening data. Table 2 shows a summary of the datasets used for this study. For four of the primary screening bioassays where there are corresponding confirmatory results, datasets have been created where the false positives from the primary screen are relabelled as Inactive. For the smaller confirmatory bioassay datasets, two types of data representation are used in order to see if adding more information improves the classification results.

**Table 2:** Summary of Bioassay datasets used in the predictive models

Assay	No of Attributes	Screening Type	Compounds	Minority Class %
AID362	144	Primary	4279	1.4%
AID604	154	Primary	59788	0.35%
AID456	153	Primary	9982	0.27%
AID688	154	Primary	27198	0.91%
AID373	154	Primary	59788	0.1%
AID746	154	Primary	59788	0.61%
AID687	153	Primary	33067	0.28%
AID746&AID1284	154	Primary and Confirmatory	59784	0.1%
AID604&AID644	154	Primary and Confirmatory	59782	0.11%
AID373&AID439	154	Primary and Confirmatory	59795	0.02%
AID687&AID721	153	Primary and Confirmatory	33046	0.06%
AID1608	154	Confirmatory	1033	6.58%
AID644	100	Confirmatory	206	32.52%
AID1284	103	Confirmatory	362	15.75%
AID439	81	Confirmatory	69	18.84%
AID721	87	Confirmatory	94	22.34%
AID1608	914	Confirmatory	1033	6.58%
AID644	914	Confirmatory	206	32.52%
AID1284	914	Confirmatory	362	15.75%
AID439	914	Confirmatory	69	18.84%
AID721	914	Confirmatory	94	22.34%

Further information on these assays may be found in the Experimental section and on the PubChem website. The AID number may be used as the search criterion.

### Data Pre-processing

The chemical structures from PubChem were downloaded in Structure Data Format (SDF) and imported into the molecular descriptor generator PowerMV [11]. A total of 179 descriptors were generated for each compound. The details of the descriptors may be found in the Experimental section. The bit-string fingerprint descriptor values that only had one value throughout the dataset (For example, all 0 s or all 1 s) were removed. For a secondary analysis, 735 additional fragment-pair fingerprint descriptors were generated for the confirmatory bioassay datasets.

### Cost-Sensitive Classifiers

Most classifiers assume equal weighting of the classes in terms of both the number of instances and the level of importance - misclassifying class A has the same importance as misclassifying class B. However, when trying to predict a minority class in an imbalanced dataset or when a false negative is deemed more important than a false positive, standard data mining techniques are not successful.

This type of problem led to the introduction of cost-sensitive classifiers where instances are predicted to have the class that has the lowest expected cost [12,13]. In Weka, two methods are used to introduce cost-sensitivity - the reweighting of the training instances according to the total cost assigned to each class in the cost matrix or predicting the class with the minimum expected misclassification cost using the values in the cost matrix. A cost matrix may be seen as an overlay to the standard confusion matrix used to evaluate the results of a predictive modelling experiment. The four sections of a confusion matrix are True Positives (TP) - in our case Active compounds correctly classified as Active; False Positives (FP) - Inactive compounds incorrectly classified as Active; True Negatives (TN) - Inactive compounds correctly classified as Inactive; False Negatives (FN) - Active compounds incorrectly classified as Inactive. For bioassay data and more importantly for screening compound selection, it is better to minimise the False Negatives at the expense of increasing the number of False Positives. One of the advantages of using cost-sensitive classifiers is that the number of False Positives may be controlled - increasing the misclassification cost of the False Negatives will potentially increase both the number of False Positives and the number of True Positives. Table 3 shows a cost matrix when there is no penalty or cost for classifying the instances correctly, a cost of 1 for misclassifying an Inactive compound (False Positive) and a cost of 5 for misclassifying an Active compound (False Negative). This means that it is more costly misclassifying the positives than misclassifying the negatives. This misclassification cost is then used to build the predictive models.

**Table 3:** A typical Cost Matrix which shows the misclassification cost for Positives and Negatives

	Actual Positive	Actual Negative
**Predicted Positive**	0 TP	1 FP

**Predicted Negative**	5 FN	0 TN

One of the problems of cost-sensitive classifiers is that there are no standards or guidelines for setting the misclassification costs. Previous research has used the ratio of positives to negatives as the misclassification cost for fraud detection [14] and for medical data classification [15]. Other research has employed the number of majority class instances as the misclassification cost [16] or bespoke methods of cost calculation that work for specific classifiers only [17]. However, when using Weka the differing data mining algorithms utilise costs differently depending on the underlying probability handling of the algorithm. For example, in Sheng and Ling [16] they have used Weka's cost-sensitive classifiers to evaluate their novel method. However, for the differing classifiers they have used across-the-board costs of 2, 5, 10 etc. This research shows that setting the Weka cost matrix is dependent on the base classifier used. For example, in one of our experiments using a cost-sensitive Naive Bayes classifier requires a misclassification cost of 2 to achieve the same results as a cost-sensitive Random Forest with a misclassification cost of 75. One of the difficulties in setting up the Weka cost matrix is that the costs are not a straight-forward ratio. Weka normalises (reweights) the cost matrix to ensure that the sum of the costs equals the total amount of instances.

For our set of experiments, we used incremental costing where the cost was increased in stages from 2 to 1000000. The misclassification cost was incremented until a 20% False Positive rate was reached - a 20% False Positive rate seemed an appropriate place to stop. This meant over 5000 classifiers were built for this study so that we could find an optimal Weka misclassification cost setting for a specific base classifier when applied to a specific type of dataset. The base classifiers used were Naive Bayes, Random Forest and Weka's implementation of a Support Vector Machine (SMO) and a C4.5 (J48) decision tree. Default Weka options were used for the Naive Bayes and Random Forest but for the SMO "build logistic models" was set to true and for the J48 tree "Pruning" was disabled. The standard cost-sensitive classifier was used for Naive Bayes, SMO and Random Forest. For J48, a bagged (Bootstrap Aggregating) meta-learner MetaCost was used as it works more efficiently for unstable, unpruned decision trees [18]. Further details of these models may be found in the Experimental section.

There are two main goals of the classification experiments - to find the most robust and versatile classifier for imbalanced bioassay data and to find out the optimal misclassification cost setting for a classifier. Even though we do not recommend using primary screening data, we have included this type of data as it tends to be larger and more imbalanced than some confirmatory screening data. These experiments are more of a survey of the classifiers rather than an experiment to gain insightful information about potential drugs for the particular targets. The datasets were randomly split into an 80% training and validation set and a 20% independent test set. To train the models cross-validation was employed. Cross-validation is a standard statistical technique where the training and validation data set is split into several parts of equal size, for example 10% of the compounds for a 10 fold cross-validation. For each run of the classifier, 10% of the data is excluded from the training set and put in a corresponding validation set. A 5 fold cross-validation was used for the training and validation of the larger datasets and a 10 fold classification for the smaller confirmatory datasets. In both cases, the resulting model from the cross-validation was applied to the test set. All reported results are based on the independent testing and not on the training.

## Results

This section first looks at the setting of the Weka cost matrix and compares the misclassification costs needed for each classifier for each dataset. We then look at the performance results of the primary screen bioassay datasets when constrained to a maximum False Positive limit of approximately 20%. The results of the two types of confirmatory bioassay datasets are then analysed and finally a comparison is made of the results of the datasets that have mixed primary and confirmatory data.

### Weka Cost Matrix

The set of experiments carried out show that there is a large variability in how the differing classifiers respond to the misclassification costs in the Weka Cost Matrix. The Naive Bayes classifier in all instances requires a smaller misclassification cost setting than the other classifiers. A Random Forest classifier requires more memory than the other classifiers, though this will be due to the fact it utilises bagging. When it could be run, the Random Forest classifier requires a large cost setting to achieve the same results as the others. Table 4 shows the Weka Cost matrix misclassification costs for the False Negatives in order to achieve the maximum number of True Positives with a False Positive rate of fewer than 20% for each classifier. The number in brackets after the dataset name is the misclassification cost if the ratio of active compounds to inactive compounds (inactives/actives) had been used.

**Table 4:** Misclassification Costs per primary screen dataset and mixed primary/confirmatory datasets

Dataset	Naive Bayes	SMO	Random Forest	J48
AID362 **(70)**	40	150	3000	285
AID604 **(281)**	40	250	Out of memory	650
AID456 **(369)**	18	200	100000	1000
AID688 **(109)**	34	78	Out of memory	220
AID373 **(963)**	20	2000	Out of memory	3000
AID746 **(162)**	25	100	Out of memory	450
AID687 **(351)**	50	250	Out of memory	680
AID746&AID1284 **(1048)**	100	1000	Out of memory	1900
AID604&AID644 **(891)**	70	750	Out of memory	1500
AID373&AID439 **(4599)**	70	9000	Out of memory	9500
AID687&AID721 **(351)**	700	6702	Out of memory	1900

In 10 out of 11 experiments, Naive Bayes has the smallest cost setting, then the SMO and finally the J48. In all instances, the performance of the classifiers would have been reduced if minority class ratios had been used as the Weka misclassification cost - there are significant differences between the optimal cost and the class ratio cost.

Confirmatory bioassay data tend to be smaller and less imbalanced (smaller Inactive/Active ratios) than primary bioassay data. For these datasets, standard classifiers were applied first (no misclassification costs) and if there was less than a 20% False Positive rate then cost-sensitive classifiers were used. Table 5 shows the misclassification costs, if any, used for the confirmatory datasets. A * indicates that the best results that could be achieved had a greater than 20% False Positive rate. The 'a' after the dataset name represents the smaller dataset and the 'b' represents the larger version of the dataset. The number in brackets after the dataset name is the misclassification cost if the ratio of active compounds to inactive compounds (inactives/actives) had been used.

**Table 5:** Misclassification Costs for False Negatives per confirmatory dataset

Dataset	Naive Bayes	SMO	Random Forest	J48
AID1608a **(14)**	2	5	75	25
AID644a **(2)**	None*	None	None	None*
AID1284a **(5)**	None*	2.7	8	2
AID439a **(4)**	None*	None	None	None
AID721a **(3)**	None*	None	None	None
AID1608b **(14)**	None	200	75	30
AID644b **(2)**	None*	None	1.5	2
AID1284b **(5)**	None*	6	8	2
AID439b **(4)**	None	None	3	None*
AID721b **(3)**	None	None	None	None

Once again, it seems that there is no connection between the ratios of Inactives:Actives to the Weka cost matrix setting. The Naive Bayes classifier has not needed any misclassification costs for 90% of the datasets, however in 60% of the datasets there are greater than 20% False Positives. Adding approximately 800 more attributes to the larger 'b' datasets has not had an effect on the setting of the misclassification costs. This illustrates that the setting of the Weka misclassification cost is arbitrary and more closely linked to the base classifier used than the class ratios or the number of attributes. As the costs we are discussing are the actual settings of the Weka cost matrix rather than class ratios, the comparison of classifiers cannot be compared using cost curves [13].

### Primary Screen Bioassay Datasets

**Figure 1:**
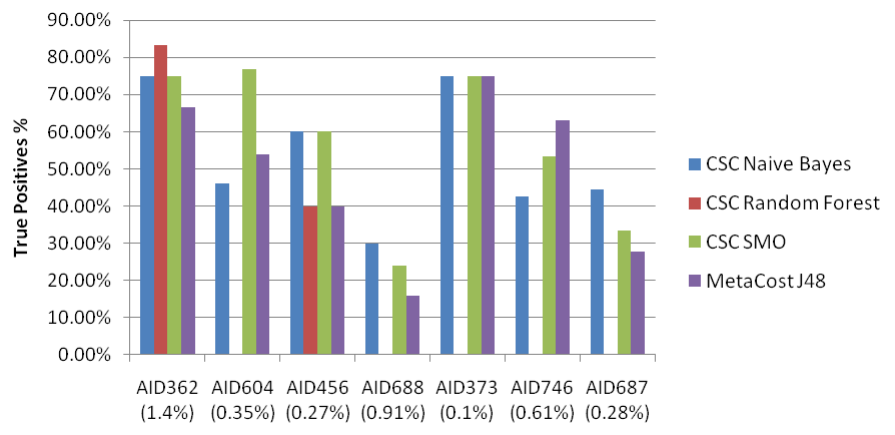
**Primary Screen datasets: True Positive rate with under or approximately a 20% False Positive rate**. The True Positive rate achieved by each type of classifier for the Primary Screen datasets. A maximum limit of 20% False Positives were allowed.

The independent test performance of each classifier was compared by the maximum number of True Positives that could be attained with approximately a 20% False Positive rate. As mentioned previously, one of the advantages of using cost-sensitive classifiers is that the False Positive rate may be controlled. Figure 1 shows the True Positive rate achieved by each classifier with under a 20% False Positive rate when the training models were applied to the independent test set. The minority class % of each dataset is shown in brackets.

These figures are quite promising considering the degree of imbalance in the bioassay data. Though classifier accuracy and precision are not the best statistical evaluation methods for imbalanced datasets, the results of these may be found in the supplementary Excel results file. See Additional file 1: Full results of the classification experiments.

Some observations from the experiments are detailed below:

• Even though all the datasets are from primary screening bioassays, there is a big difference in classifier performance. There appears to be no relation of performance to the number of compounds in the bioassay or the size of the minority class. From a cost-sensitive classifier point of view, the experiments show that these types of classifiers are capable of producing some good True Positive rates with a controllable False Positive rate for highly imbalanced data. From a bioassay point of view, it is questionable how helpful these models are: primary screening usually involves a large amount of false positives. For example, AID688 had a 100% false positive rate and AID373 had a 90% false positive rate. This leads back to the issue of whether this type of data should be used for virtual screening.

• As a Random Forest classifier is an ensemble classifier (an ensemble of Random Trees), it requires more computational memory than the other classifiers. It has not been able to run when there has been over 27,000 compounds

• Overall, Weka's implementation of the cost-sensitive Support Vector Machine, the SMO, has performed consistently well. A disadvantage of the SMO has been the amount of time taken to build the model and run the 5 fold cross-validation - in some cases the model took 7 hours to complete per cost setting used. The cost-sensitive Naive Bayes models were the quickest to build and the J48 and Random Forest models took, on average, about 1 hour per cost-setting to build.

### Confirmatory Screen Bioassay Datasets

The independent test performance of each classifier has been harder to compare as some classifiers could not achieve fewer than 20% False Positives. The compounds in confirmatory bioassay data (ie. those compounds that were deemed Active in the primary screen) are, in general, quite similar in terms of unique attributes. In some cases, there has been a 50% reduction in the fingerprint data representation when these attributes are removed. For this reason, another set of experiments was carried out where more descriptors were generated. This could not be done with the primary screening datasets because of computational memory limitations. Though these types of datasets are relatively small with only a small imbalance of Actives and Inactives, the classifiers have not been very successful at predicting the bioassay's active compounds. This could be due to the fact that the compounds in a confirmatory screen are usually closer in structure and physicochemical properties. Table 6 shows the results of both sets of experiments in terms of the True Positive and False Positive rates. The 'a' after the dataset name represents the smaller dataset and the 'b' represents the larger version of the dataset. The numbers of attributes in the datasets are written in brackets after the dataset name.

**Table 6:** The True Positive and False Positive rates for the confirmatory bioassay datasets

Dataset	Naive Bayes		SMO		Random Forest		J48	
	TP%	FP%	TP%	FP%	TP%	FP%	TP%	FP%
AID1608a (154)	23.08	19.17	30.77	8.81	30.77	8.29	15.78	20.21
AID644a (100)	38.46	39.29	23.08	17.86	23.08	7.14	38.46	28.57
AID1284a (103)	27.27	26.23	36.36	13.11	45.45	18.03	54.55	13.11
AID439a (81)	100.00	27.27	50.00	9.09	50.00	18.18	50.00	18.18
AID721a (87)	0.00	28.57	0.00	14.29	0.00	21.43	0.00	14.29
AID1608b (914)	30.77	12.95	38.46	13.47	30.77	12.95	38.46	18.13
AID644b (914)	30.77	28.57	53.85	14.29	38.46	17.86	30.77	14.29
AID1284b (914)	36.36	24.59	36.36	13.11	54.55	18.03	45.45	16.39
AID439b (914)	50.00	18.18	50.00	9.09	50.00	18.18	0.00	27.27
AID721b (914)	0.00	14.29	0.00	21.43	0.00	7.14	0.00	0.00

The results have been disappointing and the best True Positive rate that can be achieved with under a 20% False Positive rate is approximately 55% - this is worse than for the large, highly imbalanced data. The number of compounds correctly classified as Active could have been improved if the False Positive rate was increased, but it was decided that the same benchmark as the larger datasets should be used. These results raise the question of molecular structure representation - are Boolean fingerprints the best data representation? The confirmatory datasets represented with significantly more descriptors have only produced slightly better results than the smaller datasets.

### Primary/Confirmatory Screen Bioassay Datasets

**Figure 2:**
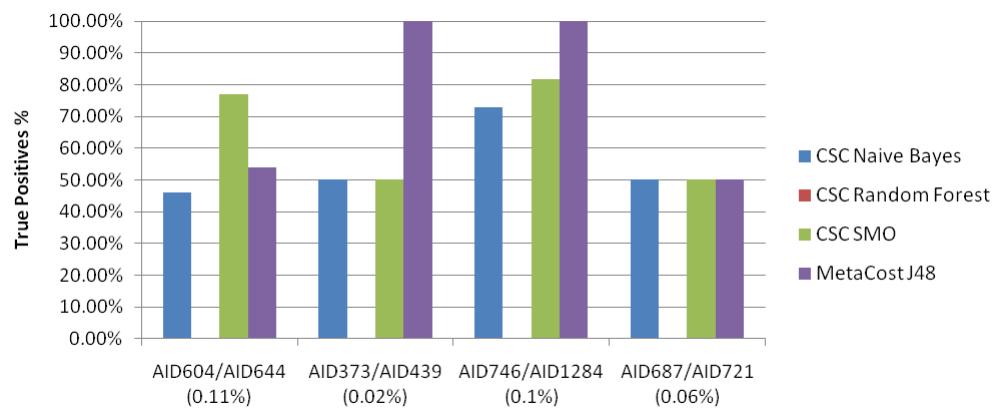
**Mixed datasets: True Positive rate with under or approximately a 20% False Positive rate**. The True Positive rate achieved by each type of classifier for the Mixed Primary Screen/Confirmatory Screen datasets. A maximum limit of 20% False Positives were allowed.

These datasets are a mixture of primary and confirmatory bioassay data - all the false positives from the primary screen are relabelled as inactive. The datasets are generally the same size as for the primary screen datasets but have a smaller minority class. Figure 2 shows the True Positive rate achieved by each classifier with under a 20% False Positive rate when the training models were applied to the independent test set. The minority class % of each dataset is shown in brackets.

These results were quite surprising - in two cases the MetaCost J48 classified all the Active compounds correctly in the independent test set with fewer than 20% False Positives. Considering the minority classes were less than 1%, this is very promising. In all cases, the datasets were too large for a cost-sensitive Random Forest to be run. The results of the mixed bioassay data were compared to the classification results of the corresponding primary and confirmatory data. Table 7 shows the bioassay datasets with the results of the best classification model highlighted.

**Table 7:** Best classification models for the bioassays with mixed, primary and confirmatory data

Assay	Best Model	TP%	FP%	Accuracy %
AID604	CSC SMO	64.29	20.59	79.36
AID644b	SMO	53.85	14.29	75.61
**AID604&AID644**	**CSC SMO**	**76.92**	**11.91**	**88.08**

AID373	MetaCost J48	75.00	14.50	85.49
AID439ab	SMO	50.00	9.09	84.62
**AID373&AID439**	**MetaCost J48**	**100.00**	**17.65**	**82.35**

AID746	MetaCost J48	63.01	20.30	79.60
AID1284b	CSC Random Forest	54.55	18.03	77.78
**AID746&AID1284**	**MetaCost J48**	**100.00**	**19.34**	**80.68**

AID687	CSC Naive Bayes	44.44	18.97	80.93
AID721b	J48	0.00	0.00	77.78
**AID687&AID721**	**MetaCost J48**	**50.00**	**9.49**	**90.49**

Interestingly, in all cases the best model, in terms of correctly classified Active compounds, has been the mixed datasets that have the smallest minority classes. This once again leads back to the question of whether primary screening data should be solely used to build bioassay predictive models - better models may be built using the confirmed Active compounds only. It is unfortunate that the models that have been the most successful are based on the hardest to obtain data from PubChem.

## Conclusions and Discussion

This paper has examined the three main problems associated with the virtual screening of bioassay data - the access to freely-available curated data, the number of false positives that occur in the primary screening process and the imbalance of Active compounds to Inactive compounds. Though the first two of these problems are not solvable by this research, it is still important that these problems are pointed out to researchers of virtual screening. This research has shown that the bioassay data at PubChem is not recorded in a standard and consistent way and some entries contain erroneous information. It is unfortunate that there is no direct search facility where related primary and confirmatory bioassays may be retrieved together - the classification models that have been the most successful are based on the hardest to obtain data from PubChem. The number of false positives from the HTS primary screen process is very high and maybe virtual screening techniques should be applied to the bioassays where there is corresponding confirmatory data.

Weka is a tool that is used by the academic community for both primary and comparative studies and it is important to explain how the cost-sensitive classifiers handle misclassification costs. Previous research has used across-the-board cost settings for differing classifiers and this research has shown that this is not the best way to implement cost-sensitivity in Weka. When using Weka, the cost matrix should be set according to the classifier being used rather than to the ratio of the minority class. From the survey of cost-sensitive classifiers carried out, the Support Vector Machine (SMO) and C4.5 decision tree learner (J48) have performed quite well considering the sizes of the minority classes. The poor results from the confirmatory bioassay experiments have led to a question of molecular structure data representation and this is an area for future work.

For the virtual screening of bioassay data, it is recommended that both primary and the corresponding confirmatory screening data are used.

## Experimental

### Bioassay Datasets

The following are the descriptions of the datasets used for these experiments. See additional files Additional file 2: Training and Testing primary screen datasets in CSV format.

Additional file 3: Training and Testing primary screen datasets in CSV format.

Additional file 4: Training and Testing confirmatory screen datasets in CSV format.

Additional file 5: Training and Testing primary/confirmatory screen datasets in CSV format.

• **AID362 **details the results of a primary screening bioassay for Formylpeptide Receptor Ligand Binding University from the New Mexico Center for Molecular Discovery. It is a relatively small dataset with 4279 compounds and with a ratio of 1 active to 70 inactive compounds (1.4% minority class). The compounds were selected on the basis of preliminary virtual screening of approximately 480,000 drug-like small molecules from Chemical Diversity Laboratories.

• **AID456 **is a primary screen assay from the Burnham Center for Chemical Genomics for inhibition of TNFa induced VCAM-1 cell surface expression and consists of 9,982 compounds with a ratio of 1 active compound to 368 inactive compounds (0.27% minority). The compounds have been selected for their known drug-like properties and 9,431 meet the Rule of 5 [19].

• **AID688 **is the result of a primary screen for Yeast eIF2B from the Penn Center for Molecular Discovery and contains activity information of 27,198 compounds with a ratio of 1 active compound to 108 inactive compounds (0.91% minority). The screen is a reporter-gene assay and 25,656 of the compounds have known drug-like properties.

• **AID604 **is a primary screening bioassay for Rho kinase 2 inhibitors from the Scripps Research Institute Molecular Screening Center. The bioassay contains activity information of 59,788 compounds with a ratio of 1 active compound to 281 inactive compounds (1.4%). 57,546 of the compounds have known drug-like properties.

• **AID373 **is a primary screen from the Scripps Research Institute Molecular Screening Center for endothelial differentiation, sphingolipid G-protein-coupled receptor, 3. 59,788 compounds were screened with a ratio of 1 active compound to 963 inactive compounds (0.1%). 57,546 of the compounds screened had known drug-like properties.

• **AID746 **is a primary screen from the Scripps Research Institute Molecular Screening Center for Mitogen-activated protein kinase. 59,788 compounds were screened with a ratio of 1 active compound to 162 inactive compounds (0.61%). 57,546 of the compounds screened had known drug-like properties.

• **AID687 **is the result of a primary screen for coagulation factor XI from the Penn Center for Molecular Discovery and contains activity information of 33,067 compounds with a ratio of 1 active compound to 350 inactive compounds (0.28% minority). 30,353 of the compounds screened had known drug-like properties.

• **AID1608 **is a different type of screening assay that was used to identify compounds that prevent HttQ103-induced cell death. National Institute of Neurological Disorders and Stroke Approved Drug Program. The compounds that prevent a release of a certain chemical into the growth medium are labelled as active and the remaining compounds are labelled as having inconclusive activity. AID1608 is a small dataset with 1,033 compounds and a ratio of 1 active to 14 inconclusive compounds (6.58% minority class).

• **AID644 **confirmatory screen of AID604

• **AID1284 **confirmatory screen of AID746

• **AID439 **confirmatory screen of AID373

• **AID721 **confirmatory screen of AID746

### Bioassay Descriptors

As previously mentioned, the software PowerMV [11] was used to generate descriptors for the bioassay SDF files from PubChem. 179 descriptors were generated for each dataset.

• 8 descriptors useful for characterizing the drug-likeness of a compound. These include XlogP (the propensity of a molecule to partition into water or oil), the number of Hydrogen bond donors and acceptors, molecular weight, polar surface area, the number of rotatable bonds, a descriptor to indicate if the compound penetrates the blood-brain barrier and a descriptor for the number of reactive or toxic functional groups in the compound.

• 24 continuous descriptors based on a variation of BCUT descriptors to define a low dimensional chemistry space. The method used by PowerMV differs from BCUT in that PowerMV uses electro-negativity, Gasteiger partial charge or XLogP on the diagonal of the Burden connectivity matrix before calculating the eigenvalues.

• 147 bit-string structural descriptors known as Pharmacophore Fingerprints based on bioisosteric principles - two atoms or functional groups that have approximately the same biological activity are assigned the same class.

For the confirmatory datasets, Fragment Pair Fingerprints were also generated using PowerMV. For fragment-based descriptors, 14 classes of paired functional groups are defined. For example, two phenyl rings separated by two bonds are expressed as AR_02_AR [11].

### Meta-Learners and Base Classifiers

The following classifiers were implemented for this research. Please note that italics represent Weka key words so that the experiments may be reproducible.

• Weka's *CostSensitiveClassifier *was used for the base classifiers Naive Bayes, SMO and Random Forest. Cost-sensitivity can be achieved in two ways - the reweighting of the training instances according to the total cost assigned to each class or predicting the class with the minimum expected misclassification cost. The former was used for this research and therefore the *MinimizeExpectedCost *option was set to *False*. Our preliminary experiments, not documented here, showed that the standard *CostSensitiveClassifier *produced better results for these base classifiers than the meta-learners *AdaBoost *and *MetaCost*.

• *MetaCost *combines the predictive benefits of bagging (combining the decisions of different models) with a minimized expected cost model for cost-sensitive prediction. An ensemble classifier is built using bagging and it is used to relabel the training data based on the minimised expected costs [6]. *Metacost *works well with unstable models and our preliminary experiments found that using *Metacost *with the *J48 *unpruned tree produced better results than *AdaBoost *and *CostSensitiveClassifier*.

• *NaiveBayes *is a probabilistic classifier based on applying Bayes' theorem with strong independence assumptions. A Naive Bayes classifier assumes that the presence or absence of a particular feature of a class is unrelated to the presence or absence of any other feature. The Weka defaults for this classifier were used.

• *SMO *is Weka's implementation of the Support Vector Machine where the sequential minimal optimisation algorithm is used to train a support vector classifier. With the SMO, linear models may be used to implement non-linear class boundaries. As the meta-learner *CostSensitiveClassifier *works better with probability estimates, the SMO option *BuildLogisticModels *was set to *True*.

• *J48 *is Weka's implementation of a C4.5 decision tree learner. *J48 *was used for these experiments as it is not a black box approach and may provide added value to the classification tasks. *MetaCost *works better with unstable data and therefore the *J48 *option *Unpruned *was set to *True*.

• *RandomForest *is an ensemble classifier that consists of many *RandomTrees*, in this case 10. The output of the *RandomForest *is the class that is the statistical mode of the class's output by the individual trees. Weka defaults were used for the classifier. As a Random Forest classifier is a bagged classifier, more computer memory is required to run them than for the other base classifiers used.

## Abbreviations

SVM: Support Vector Machine; HTS: High-Throughput Screening; 3D: 3 Dimensional; CSC: Cost-Sensitive Classifier; CSV: Comma Separated Values; PS: Primary Screen; CS: Confirmatory Screen; SDF: Structure Data Format; SMO: Sequential Minimal Optimisation.

## Competing interests

The author declares that they have no competing interests.

## Supplementary Material

Additional file 1 **Virtual Screening Results**. Excel spreadsheet containing all the results of the classification experiments.

Additional file 2 **Virtual Screening Data Primary**. Training and Testing primary screen datasets in CSV format. Click here for file

Additional file 3 **Virtual Screening Data Primary**. Training and Testing primary screen datasets in CSV format. Click here for file

Additional file 4 **Virtual Screening Data Confirmatory**. Training and Testing confirmatory screen datasets in CSV format. Click here for file

Additional file 5 **Virtual Screening Data Mixed**. Training and Testing primary/confirmatory screen datasets in CSV format. Click here for file
